# Developing a Hetero-Alkali-Metal Chemistry of 2,2,6,6-Tetramethyl-piperidide (TMP): Stoichiometric and Structural Diversity within a Series of Lithium/Sodium, Lithium/Potassium and Sodium/Potassium TMP Compounds

**DOI:** 10.1002/chem.201101167

**Published:** 2011-07-15

**Authors:** David R Armstrong, Alan R Kennedy, Robert E Mulvey, Stuart D Robertson

**Affiliations:** [a] WestCHEM, Department of Pure and Applied Chemistry, University of StrathclydeGlasgow, G1 1XL (UK), Fax: (+44) 141-548-4787 E-mail: r.e.mulvey@strath.ac.uk

**Keywords:** aggregation, alkali metals, amides, heterometallic complexes, X-ray diffraction

## Abstract

Studied extensively in solution and in the solid state, Li(TMP) (TMP=2,2,6,6-tetramethylpiperidide) is an important utility reagent popular as a strongly basic, weakly nucleophilic tool for C–H metallation. Recently, there has been a surge in interest in mixed metal derivatives containing the bulky TMP anion. Herein, we start to develop hetero (alkali metal) TMP chemistry by reporting the *N*,*N*,*N′*,*N′*-tetramethylethylenediamine (TMEDA)-hemisolvated sodium–lithium cycloheterodimer [(tmeda)Na(μ-tmp)_2_Li], and its TMEDA-free variant [{Na(μ-tmp)Li(μ-tmp)}_∞_], which provides a rare example of a crystallographically authenticated polymeric alkali metal amide. Experimental observations suggest that the former is a kinetic intermediate en route to the latter thermodynamic product. Furthermore, a third modification, the mixed potassium–lithium-rich cycloheterotrimer [(tmeda)K(μ-tmp)Li(μ-tmp)Li(μ-tmp)], has also been synthesised and crystallographically characterised. On moving to the bulkier tridentate donor *N*,*N*,*N*′,*N*′′,*N*′′-pentamethyldiethylenediamine (PMDETA), the additional ligation forces the sodium–lithium and potassium–dilithium ring species to open giving the acyclic arc-shaped complexes [(pmdeta)Na(μ-tmp)Li(tmp)] and [(pmdeta)K(μ-tmp)Li(μ-tmp)Li(tmp)], respectively. Completing the series, the potassium–lithium and potassium–sodium derivatives [(pmdeta)K(μ-tmp)_2_M] (M=Li, Na) have also been isolated as closed structures with a distinctly asymmetric central MN_2_K ring. Collectively, these seven new bimetallic compounds display five distinct structural motifs, four of which have never hitherto been witnessed in TMP chemistry and three of which are unprecedented in the vast structural library of alkali metal amide chemistry.

## Introduction

Alkali-metal amide bases have long been at the forefront of chemical synthesis, mainly through metallation chemistry (transformation of an inert C–H bond to a labile C–metal bond), as a consequence of their strong Brønsted basicity and low nucleophilicity. Such bases can generally have their reactivity boosted by utilising a Lewis donor to decrease their state of aggregation and aid solubility. Central to understanding their reactivity lies the recognition of their structural chemistry, because knowledge of the latter can give valuable insight into the former. Although an infinite number of structural possibilities exist for a given alkali metal amide [M(NR_2_)]_*x*_(donor)_*y*_, only very few are typically seen in practice, including but not limited to cyclodimers, cyclotrimers or polymers.

**Scheme 1:**
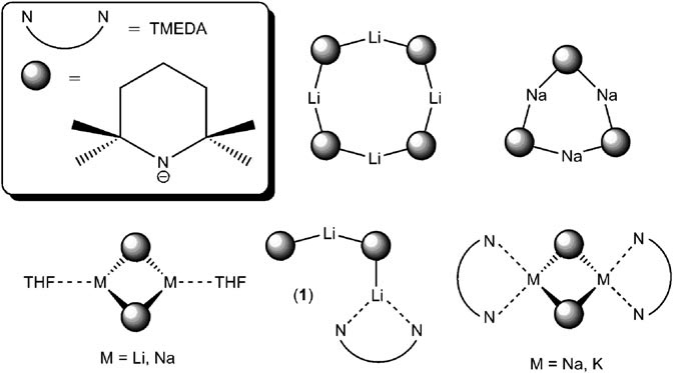
Crystallographically characterised alkali-metal–TMP complexes.

Spearheading alkali metal amide chemistry continuously for the past 40 years has been the utility base TMP (2,2,6,6-tetramethylpiperidide). These years have witnessed two distinct eras of development. Lithiation dominated the original era through utilisation of LiTMP^1^ (along with other sterically demanding lithium secondary amides, notably diisopropylamide (LDA^2^)) and this area continues to grow today. Indeed, LiTMP chemistry provides hitherto the stand-alone structure in this field—TMEDA (*N*,*N*,*N′*,*N′*-tetramethylethylenediamine)-solvated LiTMP, which adopts a unique hemisolvated “open dimer” structure,^3^ [(tmeda)Li(μ-tmp)Li(tmp)] (**1**, Scheme 1).^4^

**Scheme 2:**
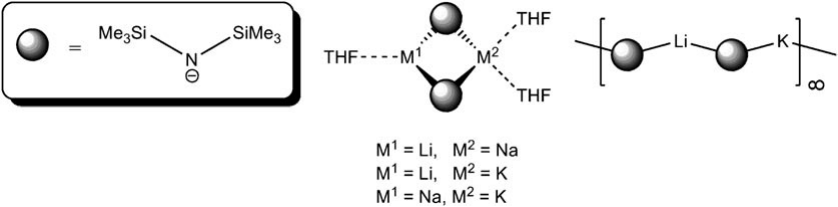
Crystallographically characterised heterometallic alkali-metal HMDS complexes.

Kick-started by Eaton et al. through the report of magnesiations of carboxamides by using (TMP)MgBr,^5^ the modern era of multicomponent TMP bases is accelerating rapidly, typified by the turbo-Hauser base [(tmp)MgCl**⋅**LiCl] developed by Knochel and co-workers.^6^ The fundamental distinction between the old and the new TMP bases is that the latter do not deprotonate by lithiation, but by magnesiation, zincation or alumination amongst others,^7^ that is, by metals that are generally less reactive than lithium but are activated to a higher reactivity through cooperative effects between the different components in their multicomponent modifications. Multicomponent TMP bases generally have an alkali-metal–non-alkali-metal (e.g., Mg, Zn, Al, Mn) combination integrated with ligands in molecular assemblies. Surprisingly, there appears to have been no progress made in trying to merge ideas from each era, that is in constructing multicomponent TMP complexes^8^ based on mixed alkali-metal–alkali-metal pairings, a fact made all the more perplexing because hetero-alkali-metal imide,^9^ alkoxide,^10^ primary amide,^11^ heteroanionic alkoxide/primary amide^12^ and other secondary amide^13^ complexes have all received attention. The excellent recent studies of O’Shea et al. have implicated TMP in a hetero-alkali-metal base for performing selective vinyl^14^ and alkyl/aryl^15^ deprotonation in a series of substituted toluenes, albeit using a heteroanionic alkyl/alkoxide/amide base. The empty file on hetero-(alkali-metal)–TMP chemistry is all the more extraordinary given the comprehensive dossier amassed for LiTMP^16^ (and to a much lesser extent for NaTMP^17^) and the fact that 20 years have passed since Williard and Nichols established a hetero-(alkali-metal) chemistry of HMDS^18^ (HMDS=1,1,1,3,3,3-hexamethyldisilazide), another utility amide though a much less powerful base than LiTMP. Williard and Nichols reported a series of THF-solvated heterodimers displaying (M^1^M^2^N) rings (M^1^=Li, M^2^=Na or K; M^1^=Na, M^2^=K; Scheme 2).

In this work, we document the beginning of hetero-(alkali-metal) TMP chemistry by reporting the synthesis and structural characterisation of seven distinct complexes. Expanding substantially upon the structurally diversity of alkali metal–TMP systems and introducing several new potential metallating agents; these results show that hetero-alkali-metal–TMP chemistry is ripe for development.

## Results and Discussion

**X-ray crystallographic study**: Our own study started with the synthesis of the TMEDA solvate of the mixed sodium–lithium complex [(tmeda)Na(μ-tmp)_2_Li] (**2**), which bears a close similarity to the HMDS heterodimers and represents the first example of a hemisolvated closed dimer. Solutions of *n*BuNa, *n*BuLi, TMP(H) and TMEDA in hexane in either a 1:1:2:2 or, matching the solvation of **2**, a 1:1:2:1 stoichiometry furnished crystals of **2**. However, the picture changed dramatically on adjusting the conditions of this reaction. Whereas introducing TMEDA last and immediately cooling the reaction mixture to −35 °C produced high yields of **2**, performing all of the procedure at ambient temperature induced the precipitation of a different solid. Insoluble in arene and aliphatic hydrocarbon solvents, this solid surprisingly did not contain any TMEDA (though its presence seemed necessary for the formation of the solid, otherwise no precipitation occurred over 6 h) as determined by NMR analysis in [D_8_]THF solution. Repeating the ambient-temperature procedure but without stirring the solution following TMEDA addition gave a crystalline form of the solid after standing overnight. X-ray crystallographic studies established its identity as the TMEDA-free mixed sodium–lithium polymer [{Na(μ-tmp)Li(μ-tmp)}_∞_] (**3**).

**Figure 1:**
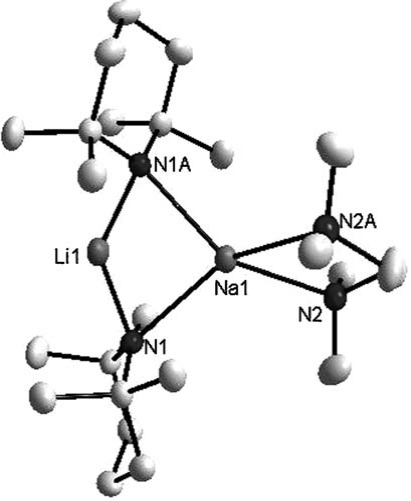
Molecular structure of **2** with hydrogen atoms omitted and thermal ellipsoids drawn at 50 % probability. The symmetry operation to generate the equivalent atoms marked A is 1.5−*x*, 0.5−*y*, *z*. Selected bond lengths [Å] and angles [^o^]: Li1–N1 1.917(4), Na1–N1 2.513(3), Na1–N2 2.535(3); N1-Li1-N1A 132.5(4), N1-Na1-N1A 88.57(11), Li1-N1-Na1 69.5(2), N2-Na1-N2A 73.13(12), N2-Na1-N1 112.23(8), N2-Na1-N1A 140.50(8).

**Scheme 3:**
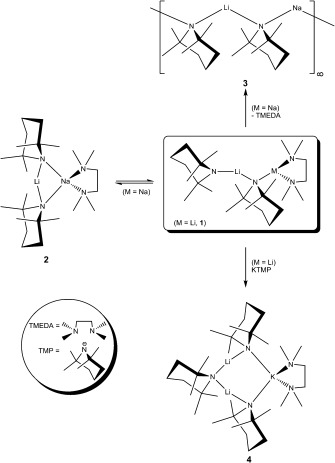
Reaction scheme to give the hetero alkali-metallic TMP species **2**, **3** and **4**.

**Figure 2:**
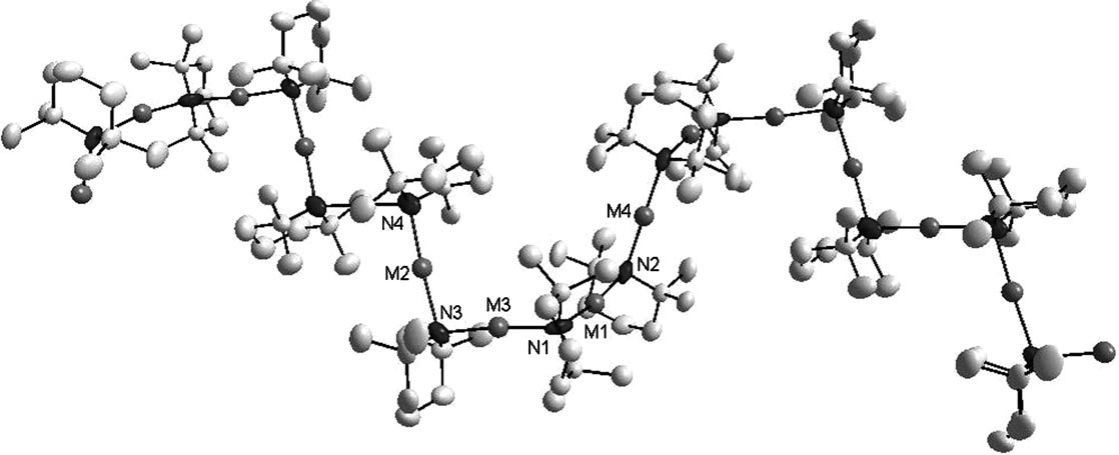
Molecular structure of **3** with hydrogen atoms omitted and thermal ellipsoids drawn at 50 % probability.

The molecular structure of the heterometallic compound **2** (Figure 1) draws an interesting contrast with the homometallic analogue [(tmeda)Li(μ-tmp)Li(tmp)] (**1**).^4^ Substituting one Li centre by a larger Na centre (thereby elongating affected metal–N bonds) enables in effect the open dimer structure of **1** (Scheme 3) to close to a (NaNLiN) ring structure in **2** with the solitary TMEDA chelated to Na. Two coordinate, the remaining Li is noticeably exposed [N1-Li1-N1A bond angle=132.5(4)°, note that the closest Li⋅⋅⋅Me contact is very long at 2.867(3) Å],^19^ whereas Na occupies a more comfortable distorted tetrahedral geometry. With a crystallographically imposed 2-fold axis running through Li1, Na1 and the midpoint of TMEDA, the TMP anions are equivalent and lie near perpendicular to the planar NaNLiN ring (the plane made up of the two Cα atoms and N1 lies 69.7(1)° to the Li1-N1-Na1 plane) adopting a chair conformation with the Cγ apex and Li *syn* with respect to the NaNLiN ring. TMEDA is also oriented perpendicular to the azabimetallic ring (the N2Na1N2A plane tilts 61.8(1)° away from the N1Na1N1A plane). The Li–N bond length (1.917(4) Å) resembles that of the terminal Li–N_TMP_ bond in **1** (1.885(5) Å) and is significantly shorter than the Li–N_TMP_ bridging bond in **1** (2.049(5) Å). There is little difference between the lengths of the Na–N_TMP_ (2.513(3) Å) and the Na–N_TMEDA_ bonds (2.535(3) Å) in **2** in contrast to that in the TMEDA solvate of sodium diisopropylamide^20^ (mean Na–N_amido_=2.447; mean Na–N_TMEDA_=2.619 Å). The precision of the structure of **3** is compromised somewhat by mutual substitution disorder between the two metals (the crystal examined had an occupancy of Na/Li=43:57 %) and disorder in the orientations of the TMP rings. That notwithstanding, its polymeric constitution is unequivocal and is clearly a manifestation of the heterometallic mixture as homometallic NaTMP is a cyclotrimer^21^ and homometallic LiTMP is a cyclotetramer.^22^ Irregularly shaped and severely buckled, the one-dimensional chain of **3** (Figure 2) made up in essence of Na-N-Li-N links, repeats itself every 16 metal–N bonds. Despite the marked aggregation state rise, this bimetallic chain arrangement maintains the two-coordinate Na, Li and TMP connectivities displayed in the solvent-free monometallic TMP molecules. Given the high steric demands of TMP, which normally limit aggregation, and the fact that individually both NaTMP and LiTMP are small oligomers, it is intriguing that mixing them together produces an unsolvated polymer, crystallographically authenticated examples of which are remarkably rare^23^ in the vast literature of lithium secondary amide chemistry.^24^ The bimetallic constitution was confirmed by ion chromatography that substantiated the presence of both lithium and sodium in the sample. This was subsequently quantified by flame atomic absorption spectroscopy that suggested a Na/Li ratio of 44:56. Although there was a noticeable error (±10 %), this, coupled with the difference in the solid-state structure to the homometallic derivatives, clearly shows that both metals are present in close to equimolar amounts rather than one of them merely being a slight impurity.

The experimental observations suggest that compound **2** represents the kinetic intermediate en route to the thermodynamic product **3**. This is consistent with the failure of **2** to give good NMR spectra in hydrocarbon media, as it quickly precipitates from the solution as polymeric **3**. Assuming closed heterodimer **2** is in equilibrium with an open form (like the open dimer **1**), an alternative, intermolecular recombination of the reactive, coordinatively unsaturated end atoms could, with concomitant decoordination of TMEDA, lead to head-to-tail polymerisation (Scheme 3).

**Figure 3:**
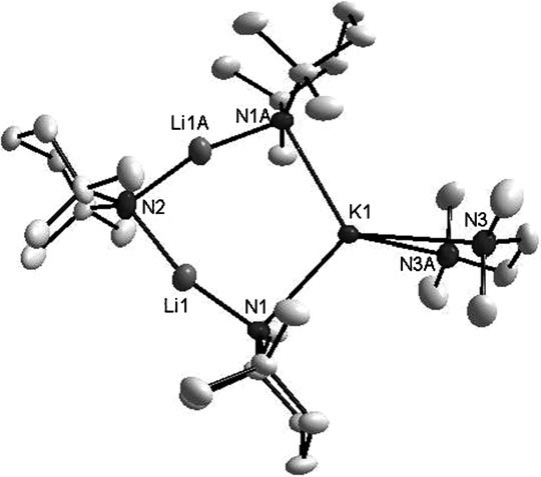
Molecular structure of **4** with hydrogen atoms and minor disordered components omitted and thermal ellipsoids drawn at 50 % probability. The symmetry operation to generate the equivalent atoms marked A is −*x*, *y*, 0.5−*z*. Selected bond lengths [Å] and angles [^o^]: K1–N1 2.890(1), K1–N3 3.016(1), Li1–N1 1.950(2), Li1–N2 1.987(2); N1-K1-N1A 107.05(3), N1-Li1-N2 164.23(14), K1-N1-Li1 97.87(8), Li1-N2-Li1A 88.75(10), N1K1-N3 119.54(3), N1A-K1-N3 122.62(3), N3-K1-N3A 59.54(3).

Mixing LiTMP with KTMP and TMEDA in hexane delivered a third variation in both stoichiometry and structure in the monopotassium–dilithium product [(tmeda)K(μ-tmp)Li(μ-tmp)Li(μ-tmp)] (**4**). This heterotrimer crystallised from solution irrespective of the K/Li starting ratio (1:1 or 1:2) employed. Drawing a similar analogy to that of **2**, the structure of **4** (Figure 3) comprising a six-membered (KNLiNLiN) ring with a single TMEDA chelating K, can be viewed as the entrapment of a KTMP monomer by the open dimer **3** accompanied by TMEDA transfer from the small to the larger, more coordinatively needy alkali metal (Scheme 3). Displaying a crystallographically imposed 2-fold axis (through N2, K1 and the midpoint of TMEDA), the molecular structure of **4** has two-coordinate near linear Li centres and a four-coordinate distorted tetrahedral K centre. The TMP chair between Li and K points its Cγ apex towards the Li side of the ring, and provides steric protection for the two coordinate metal through long-range contacts to two of its methyl groups [2.956(2) and 3.070(3) Å]. The unique TMP chair between the two Li centres is disordered about the symmetry element, pointing 50 % to one side and 50 % to the other. Unlike the TMEDA solvate **2**, compound **4** can be stirred indefinitely in hexane without precipitating a donor-free polymer, probably because the solvated six-membered ring experiences considerably less strain.

Attempts were also made to prepare a TMEDA-solvated Na/K derivative, however, despite studying reactions employing various stoichiometries the only discernible products obtained were confirmed as being the known homometallic solvates [M(tmp)(tmeda)]_2_ (M=Na, K).17e

**Figure 4:**
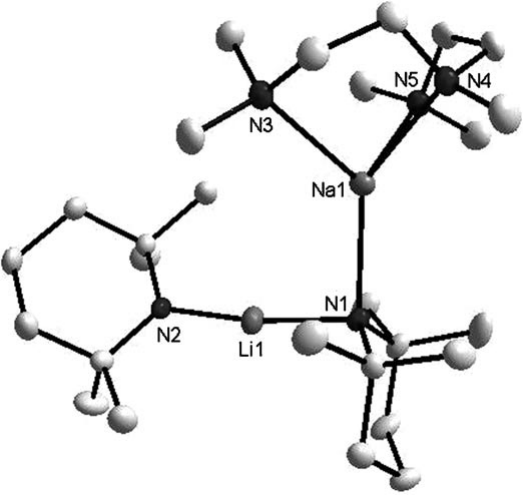
Molecular structure of **5** with hydrogen atoms omitted and thermal ellipsoids drawn at 50 % probability. Selected bond lengths [Å] and angles [^o^]: Na1–N1 2.400(2), Li1–N1 1.974(4), Li1–N2 1.858(4); Na1-N1-Li1 94.2(1), N1-Li1-N2 170.9(2).

**Figure 5:**
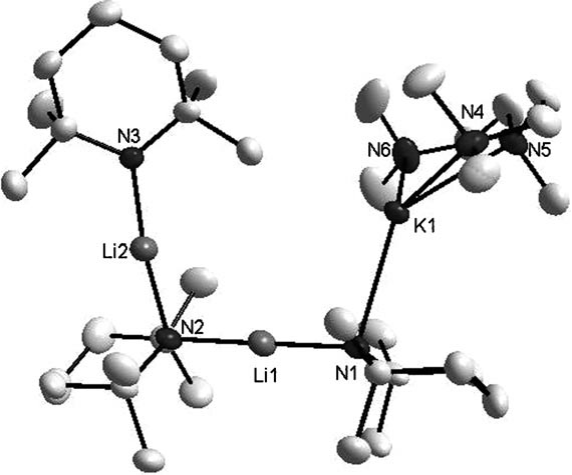
Molecular structure of **6** with hydrogen atoms and minor disordered component omitted and thermal ellipsoids drawn at 50 % probability. Selected bond lengths [Å] and angles [^o^]: K1–N1 2.842(1), Li1–N1 1.939(3), Li1–N2 1.968(3), Li2–N2 1.983(3), Li2–N3 1.856(3); K1-N1-Li1 104.2(1), N1-Li1-N2 178.2(2), Li1-N2-Li2 102.0(1), N2-Li2-N3 167.0(2).

On substituting bidentate TMEDA with the tridentate donor *N*,*N*,*N*′,*N*′′,*N*′′-pentamethyldiethylenediamine (PMDETA) the Li/Na congener followed a similar reaction pathway—upon adding a molar equivalent of the donor to the homogeneous metal/TMPH hexane solution and leaving it to stir at room temperature, precipitation of the insoluble polymer **3** was apparent, albeit over a considerably longer time (approximately 30–60 min vs. 2–5 min with TMEDA). Again, upon immediately cooling the solution, an almost quantitative crop of pale yellow crystals (**5**) grew, whose molecular structure (Figure 4) was determined. Meanwhile, addition of PMDETA to the Li/K system resulted in a brown oil separating from the hexane solution. Letting the oily mixture stand for one week at room temperature afforded a crop of X-ray-quality crystals of **6** representing a 26 % yield. Figure 5 shows the molecular structure of **6**.

The X-ray structural determinations showed that although compounds **5** and **6** have the same constitution as their TMEDA-solvated analogues **2** and **4**, that is they are hemisolvated bimetallic (LiNa or Li_2_K) molecules, one of the heavier alkali-metal–N_TMP_ bonds has cleaved to give an open acyclic structure akin to **1**, which as a consequence now loses its unprecedented status. In both cases, this change can be attributed to the extra interaction provided by the tridentate ligand PMDETA, which provides the Na/K atom with a four coordinate (N×4) environment without the need to engage a second TMP bridge. For **5**, this coordinative saturation, plus the increased steric bulk of PMDETA both play an important kinetic role as they decelerate the heads-to-tails intermolecular recombination, which is necessary to furnish polymeric **3**.

Of particular interest in the open structures of **5** and **6** are the terminal Li–N bond lengths of 1.858(4) and 1.856(3) Å, respectively, whereas the Li–N_bridging_ distances are all greater than 1.93 Å. This mirrors the bonding seen in **1**^4^ and is due to the terminal bonds being typical anion–cation bonds, whereas the longer (bridging) bonds are part of a more electron-deficient system. Like other two-coordinate N_TMP_-Li-N_TMP_ structures such as **1** (172.6(3)°) and [Li(tmp)]_4_ (168.5(4)°)^22^ these units approach linearity in **5** (170.9(2)°) and **6** (178.2(2) and 167.0(2)°). This last angle is the most removed from linearity of all those mentioned, probably in part to facilitate the close contact of a TMP methyl group to the electron-poor potassium cation (see below).

Although the TMP rings in the cyclic structures of **2** and **4** lie perpendicular to the planar LiNaN_2_ and Li_2_KN_3_ rings to allow the TMP nitrogen atoms to attain a tetrahedral geometry, the terminal TMP rings in **5** and **6** are rotated in such a way that they lie almost parallel to the pseudo-planar [MN]_*x*_ unit [the Cα-N-Cα plane lies at 32.9(1) and 17.8(1)° with respect to the MN plane in **5** and **6**, respectively]. Although the heavier alkali-metal atoms occupy a four-coordinate environment they are highly distorted from tetrahedral, because the three PMDETA nitrogen atoms all lie on one side of the metal. The terminal TMP rotation allows one of the methyl groups to maximise the steric protection afforded to the exposed face of the heavier alkali-metal atom, the methyl group lying only 3.545(2)/3.212(2) Å from Na (**5**) and K (**6**), respectively. The substantially shorter distance in **6** is attributed to the extra Li–TMP unit, which allows greater flexibility and hence a closer approach to the metal.

**Figure 6:**
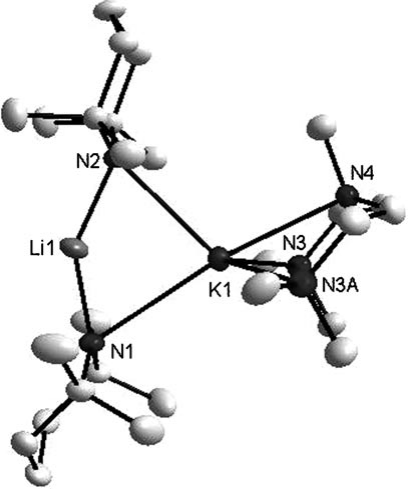
Molecular structure of **7** with hydrogen atoms omitted and thermal ellipsoids drawn at 50 % probability. The symmetry operation to generate the equivalent atoms marked A is *x*, 0.5−*y*, *z*. Selected bond lengths [Å] and angles [^o^]: Li1–N1 1.966(9), Li1–N2 2.025(7), K1–N1 3.116(2), K1–N2 2.924(2), K1–N3 2.976(2), K1–N4 2.848(2); N1-Li1-N2 144.6(5), N1-K1-N2 77.99(5), Li1-N1-K1 66.7(2), Li1-N2-K1 70.6(3).

**Figure 7:**
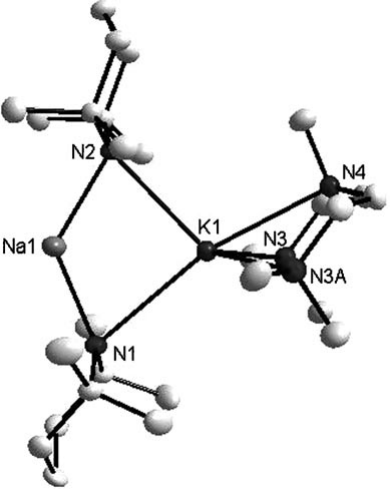
Molecular structure of **8** with hydrogen atoms omitted and thermal ellipsoids drawn at 50 % probability. The symmetry operation to generate the equivalent atoms marked A is *x*, 0.5−*y*, *z*. Selected bond lengths [Å] and angles [^o^]: Na1–N1 2.261(1), Na1–N2 2.335(1), K1–N1 3.069(1), K1–N2 2.905(1), K1–N3 3.023(1), K1–N4 2.902(1); N1-Na1-N2 126.21(1), N1-K1-N2 86.61(1), Na1-N1-K1 72.39(1), Na1-N2-K1 74.79(1).

Next, we attempted to prepare an as yet elusive trimetallic complex by mixing equimolar quantities of LiTMP, NaTMP and KTMP in hexane in the presence of PMDETA. A crop of colourless crystals resulted at −35 °C, which were shown by an X-ray structure determination to be the bimetallic 1:1 complex [(pmdeta)K(μ-tmp)_2_Li] (**7**). However, the crystal structure showed that **7** had co-crystallised with 10 % of the isostructural species [(pmdeta)K(μ-tmp)_2_Na] (**8**). Although compound **8** could be subsequently rationally synthesised from a bimetallic mixture, attempts to prepare compound **7** from only its constituent parts were unsuccessful hinting that the Li/K complex needs its Na/K congener as a template scaffold on which to crystallise. Both structures (Figures 6 and 7, respectively) fall into the category of a closed dimer as was the case with structure **2**, however their crystallographically imposed 2-fold axis runs perpendicular to that of **2**, running through the planar MN_2_K ring, N4 and the two γ-carbon atoms of the TMP rings.

**Scheme 4:**
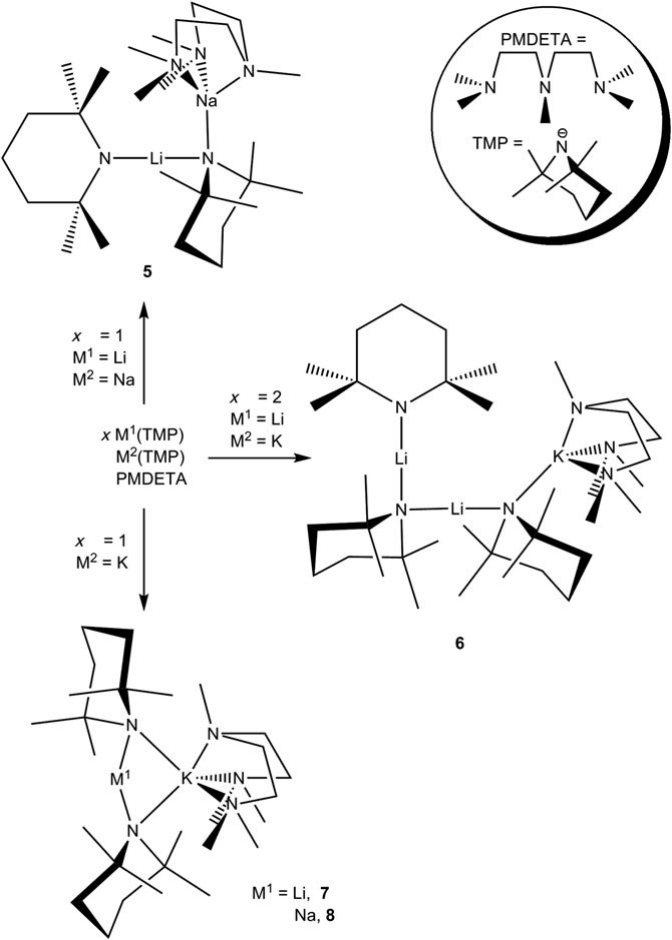
Reaction scheme to give the hetero alkali-metallic TMP species **5**–**8**.

It is conspicuous that these structures of **7** and **8** are both closed whereas the Li/Na and Li_2_/K PMDETA solvates are both open. The most striking difference between **7** and **8** and that of the other closed dimer **2** is that the TMP rings have their Cγ apices pointing in opposite directions, that is, one points towards potassium and the other towards the lighter alkali metal, whereas those in **2** both point away from potassium, which is almost certainly due to the coordination of tridentate PMDETA, with N3 and N3A lying almost directly in the M–K plane and N4 above it, forcing the methyl group on N4 to point towards a TMP ligand. This steric arrangement results in a noticeable asymmetry in the MN_2_K ring, with a shorter and longer M–N and K–N bond in the ring, with the long bonds (and consequently the short bonds) lying opposite each other. Synthetic pathways to all the heterometallic PMDETA solvates **5**–**8** are provided in Scheme 4.

The synthesis of these heterometallic TMP species can be summarised according to the general Equation (1).


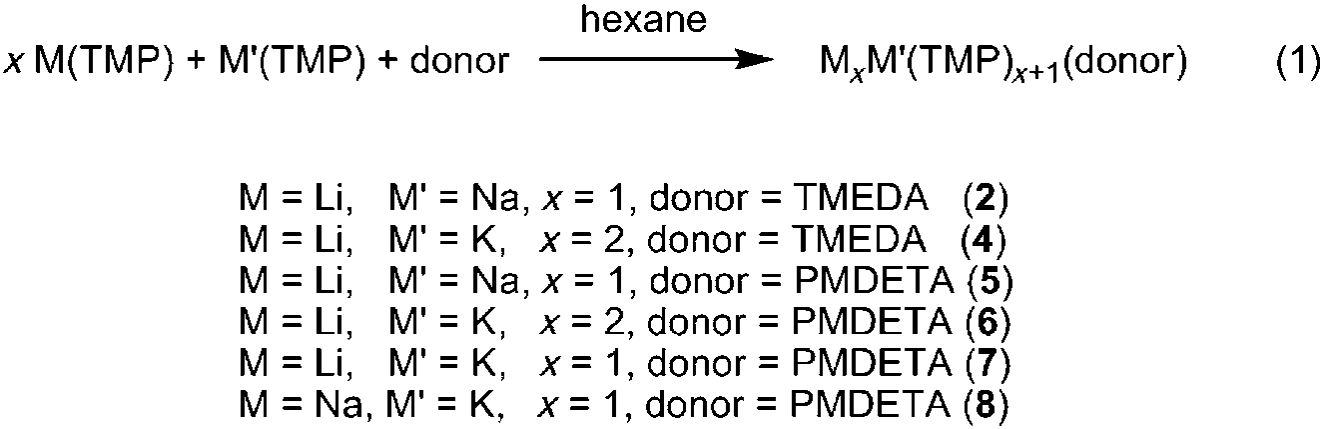
(1)

**Figure 8:**
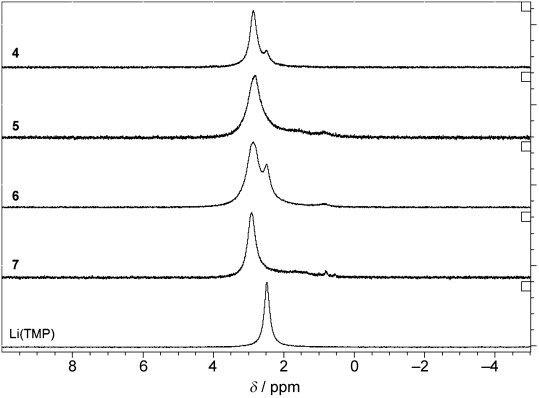
^7^Li NMR spectra of complexes **4**–**7** and Li(TMP) recorded at room temperature in C_6_D_12_.

**Solution NMR spectroscopic studies**: NMR spectral data for complexes **2** and **3** could not be obtained as soluble **2** rapidly converted to the insoluble thermodynamic product **3** on addition of non-Lewis donating NMR solvents. However, a solution of each in [D_8_]THF (which can be expected to deaggregate the structures) gave indirect evidence to support their structures, namely evidence of TMP anions in the ^1^H NMR spectrum and a strong signal in the ^7^Li NMR spectrum. The ^1^H NMR spectrum of **2** also displayed resonances corresponding to TMEDA (integration showing the 2:1 TMP/TMEDA ratio as seen in the solid state), whereas that of **3** showed no such resonances. For the more stable solvated species **4**–**8**, ^1^H NMR spectra confirmed that the TMP/donor ratio was the same as that seen in the solid state; however, non-equivalent TMP ligands could not be distinguished, even at reduced temperature or at various concentrations. More useful diagnostically were the ^7^Li NMR spectra of complexes **4**–**7** (Figure 8).

As can be clearly seen, the trimeric complexes **4** and **6** contain more than one lithium-containing species, whereas the dimeric complexes **5** and **7** consist of one principal species. A comparison with the ^7^Li NMR spectrum of homometallic Li(TMP)16f suggests that it is the minor species. In each case, the major species shows a resonance close to *δ*=3 ppm. Given that this is apparently not homometallic Li(TMP), nor a solvated derivative of Li(TMP) (as solvation typically moves the resonance upfield), we surmise that heterometallic species with unsolvated lithium centres are present in each case. The trimers would therefore appear to be in dynamic equilibrium with a dimer and Li(TMP) as shown in Equation (2).


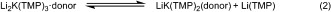
(2)

If the equilibrium in Equation (2) would lie all the way to the right [i.e., complete dissociation of the trimer into dimer and Li(TMP)] then the two peaks must be of equal intensity. As this is clearly not the case as shown in Figure 8, we suggest that the trimer resonances are in fact coincidental with the dimer resonances, which one may expect given that the lithium atoms lie in largely identical local environments regardless of whether the species is an open or closed dimer or trimer. The relative intensities of each resonance for **4** and **6** suggest that the equilibrium lies in favour of the trimer, particularly in the case of the TMEDA solvate **4**, which may be the reason why a dimeric TMEDA-solvated Li/K species cannot be isolated, even when the appropriate 1:1 stoichiometry of homometallic compounds is employed.

**Theoretical calculations**: In an attempt to shed some light on the diversity witnessed in the structures of complexes **2**–**8** we turned to DFT calculations by using the Gaussian 03 package.^25^ All complexes were modelled with the exception of **3** due to its infinite size, which makes it outside the scope of the computational methods employed. Geometry optimisation was undertaken at the HF/6-31G*^26^ level, followed by a frequency analysis. The geometry was then refined by further calculation at the B3LYP^27^/6-311G**^28^ level. The structural parameters reported were taken from the DFT calculations, whereas the total energy abstracted from the DFT calculations was adjusted by inclusion of the zero-point energy value from the HF calculation modified by the factor 0.91.

**Scheme 5:**
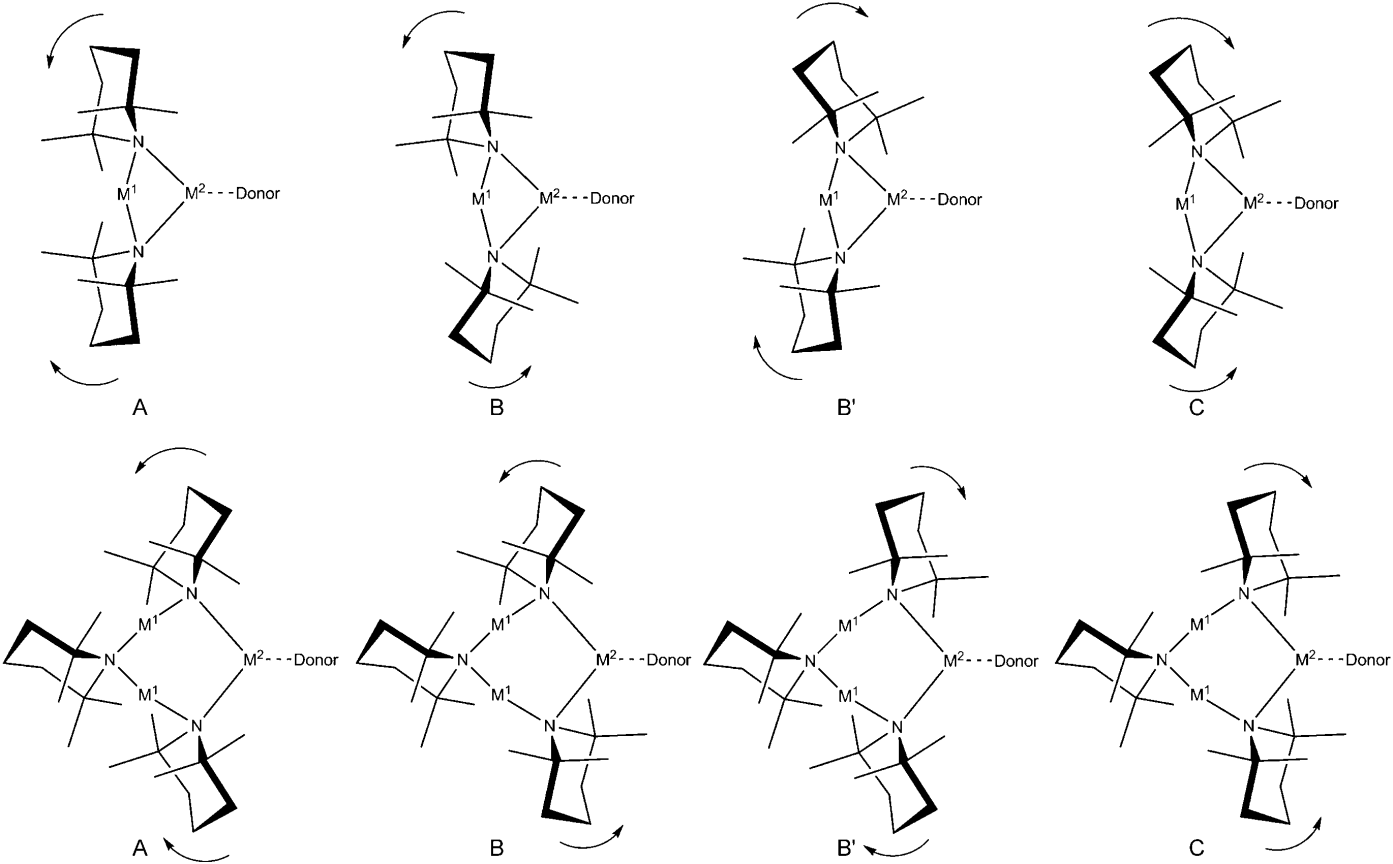
Different conformations of the TMP rings considered in the DFT study with arrows denoting directional conformation of the TMP ring.

For the TMEDA solvates, the energetically most favourable bulk nuclearity was first calculated (dimer vs. trimer, Table 1) with respect to homometallic starting materials, with the resulting energy minimum species then studied further with respect to their possible different TMP conformations. Thus the dimeric species were modelled with their TMP rings lying according to Scheme 5 (the three distinct conformations are denoted A, B and C; B=B′ when donor is TMEDA). For PMDETA species, four conformations were considered because the asymmetric chelating pattern of the tridentate donor breaks the *C*_2_ symmetry, which passes through the centre of the molecule (i.e., B≠B′). This is also the case for all trimeric species as here the unique TMP ring also breaks the aforementioned *C*_2_ symmetry. The PMDETA complexes were not subjected to energetic calculations at this juncture as the PMDETA-solvated homometallic reagents needed for comparison remain elusive.

**Table 1:** Energetics of TMEDA-solvated heterometallic species with respect to homometallic starting materials.

		Nuclearity	Relative energy [kcal mol^−1^]
Li	Na	dimer	−1.63 (=**2**_calcd_)
		trimer	−0.10
Li	K	dimer	−0.40
		trimer	−3.25 (=**4**_calcd_)
Na	K	dimer	+0.42

**Figure 9:**
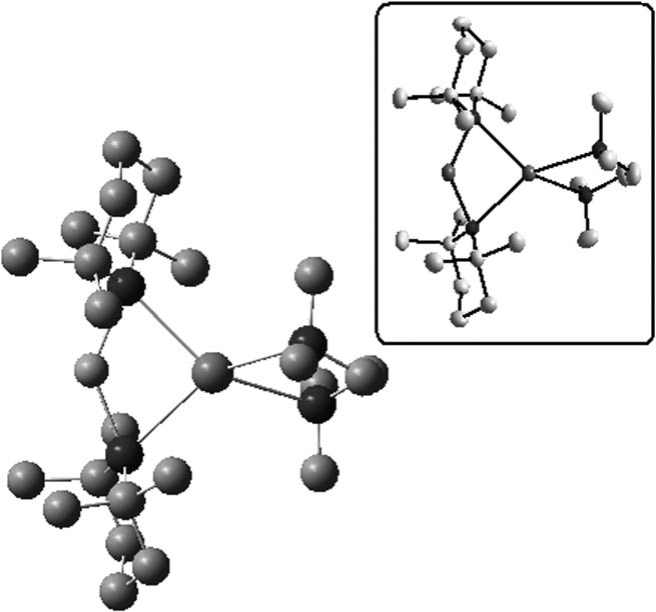
Minimum energy structure of complex **2**_calcd_ with X-ray structure (inset) for comparison.

**Figure 10:**
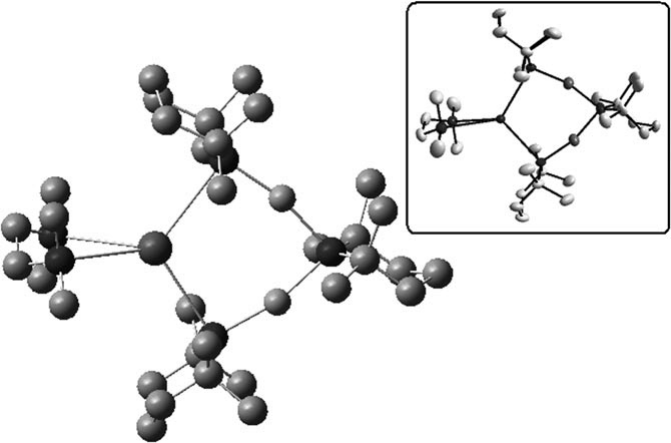
Minimum energy structure of complex **4**_calcd_ with X-ray structure (inset) for comparison.

**Figure 11:**
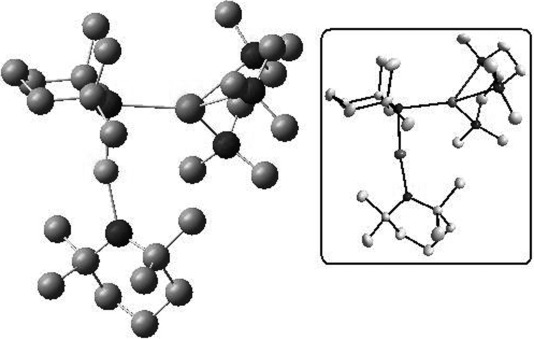
Minimum energy structure of complex **5**_calcd_ with X-ray structure (inset) for comparison.

**Figure 12:**
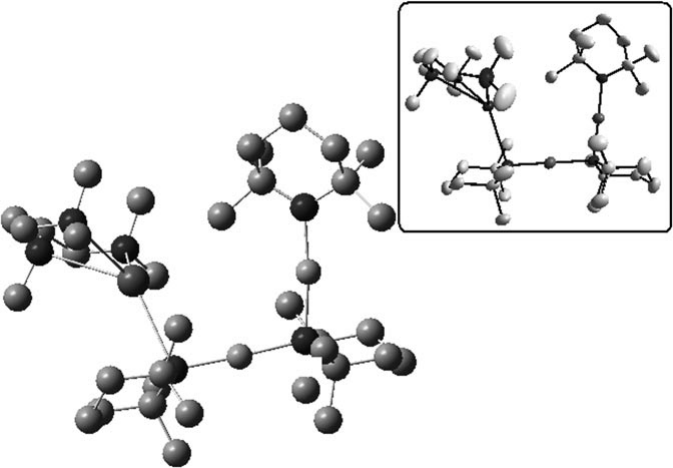
Minimum energy structure of complex **6**_calcd_ with X-ray structure (inset) for comparison.

**Figure 13:**
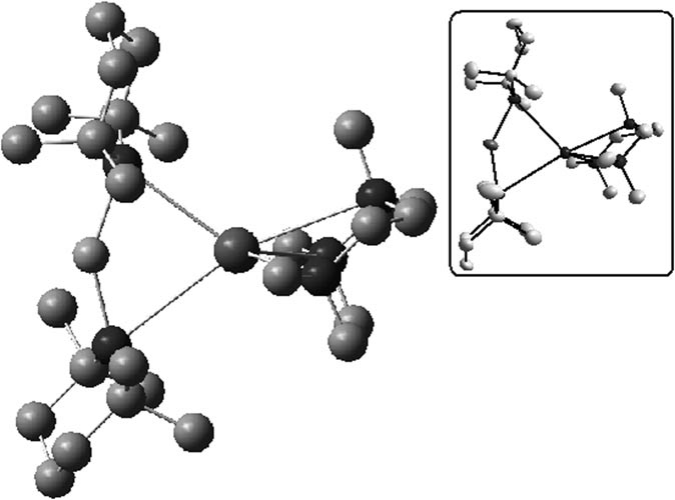
Minimum energy structure of complex **7**_calcd_ with X-ray structure (inset) for comparison.

**Figure 14:**
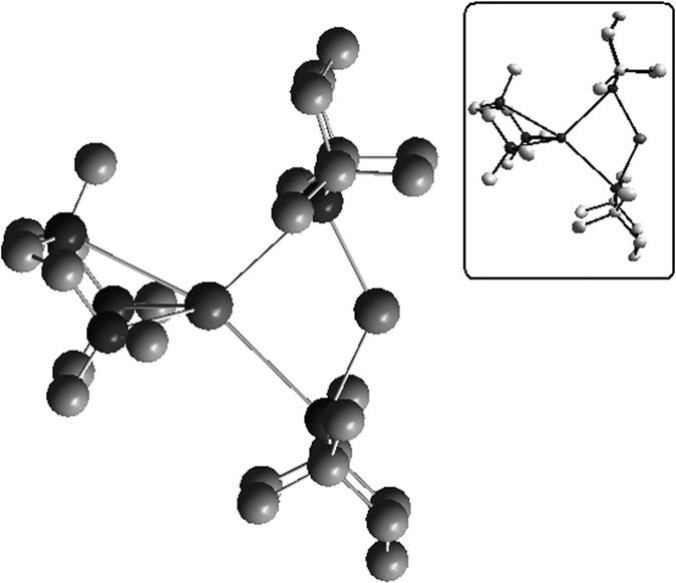
Minimum energy structure of complex **8**_calcd_ with X-ray structure (inset) for comparison.

The computed energy minimum theoretical structures in each case (**2**_calcd_, **4**_calcd_–**8**_calcd_) showed excellent correlation with the experimental molecular structures obtained through single-crystal X-ray diffraction methods (see above and Figures 9–14);^29^ in particular, the DFT calculations confirm the preferential nuclearity of the complexes (that is dinuclear for **2**_calcd_; trinuclear for **4**_calcd_). The calculations also confirm that a closed ring structure is the minimum energy conformation for **2**_calcd_, **4**_calcd_, **7**_calcd_ and **8**_calcd_ and, although the energy differences are small, it is predicted that the TMP rings will be disposed in the same positions as those seen in the X-ray-determined structures (that is type A (for **2**_calcd_ and **4**_calcd_) or type B (for **7**_calcd_ and **8**_calcd_), see Table 2). As well as predicting the type B nature of the TMP rings, the optimised structures of **7**_calcd_ and **8**_calcd_ also predict the asymmetric bonding pattern observed in the four-membered MN_2_K ring (see the Supporting Information for geometric details).

**Table 2:** Relative energies of theoretical structures 2_calcd_ and 4_calcd_–8_calcd_ in [kcal mol^−1^].

Complex	A	B	B′	C
**2**	0.00	0.76	–	1.31
**4**	0.00	1.03	5.14	3.00
**5**	2.43	0.00	0.75	4.13
**6**	1.56	0.00	1.68	1.75
**7**	0.96	0.00	0.27	0.72
**8**	2.79	0.00	0.84	0.45

The geometrical optimisations for compounds **5**_calcd_ and **6**_calcd_ commenced with closed ring structures, however, in each case as the energy minimum calculations proceeded free of constraints (in these cases through model B) they opened up into an open dimer and open trimer, respectively, again in accord with their solid-state experimental structures. As is seen in the solid state, the calculations predict the rotation of the terminal TMP ligand so that it is no longer perpendicular to the metal–nitrogen plane, with one of the methyl arms protruding toward the donor-solvated metal centre to provide steric protection, with the predicted C–metal values being 3.462 and 3.212 Å, respectively (experimentally determined values for **5** and **6** are 3.545(2) and 3.212(2) Å, respectively).

## Conclusion

**Scheme 6:**
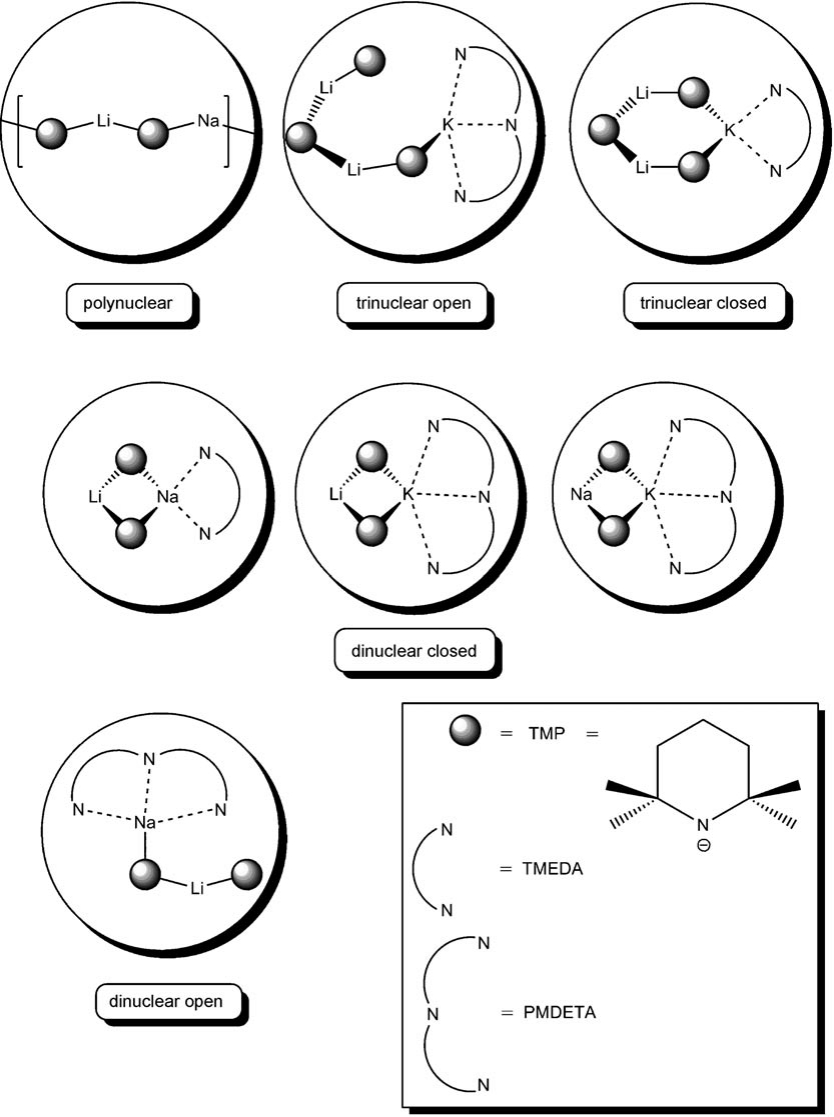
New library of hetero-alkali-metal–TMP complexes.

This study introduces the first examples of hetero-alkali-metallic complexes of one of the most important utility secondary amides—namely sterically demanding 2,2,6,6-tetramethylpiperidide (TMP). A series of seven new homoanionic compounds have been synthesised through a facile co-complexation protocol by using polydentate N-donors in non-polar medium. More than doubling the library of crystallographically characterised alkali metal–TMP complexes, seven novel structures are presented, which belong to five distinct categories; namely polynuclear, trinuclear open, trinuclear closed, dinuclear open, and dinuclear closed (see Scheme 6).

These structures are highly unusual in the vast landscape of alkali metal structural chemistry, with only one precedented example of a polynuclear hetero-alkali-metal amide in the hexamethyldisilazide [Li(hmds)K(hmds)]_∞_23b and one precedented example of a dinuclear open structure [(tmeda)Li(μ-tmp)Li(tmp)]^4^ having been reported thus far. Trinuclear-closed, trinuclear-open, and hemisolvated-dinuclear-closed structures are the first of their kind to be synthesised and crystallographically characterised. The dimeric species seem to maintain their structures in non-polar solution, whereas the trimeric species partially exclude unsolvated Li(TMP) to give a heterometallic dinuclear compound in equilibrium with the parent trinuclear compound. DFT calculations show excellent correlation with the solid-state structures. We now intend to study the synthetic utility of these new compounds and hope to make progress in PMDETA-solvated homometallic TMP chemistry with the results to be divulged in due course.

## Supplementary material

Detailed facts of importance to specialist readers are published as ”Supporting Information”. Such documents are peer-reviewed, but not copy-edited or typeset. They are made available as submitted by the authors.

## References

[1] Campbell M, Snieckus V, Paquette LA (1995). Encyclopedia of Reagents for Organic Synthesis Vol. 5.

[2] Snieckus V (1990). Chem. Rev.

[4] Williard PG, Liu Q-Y (1993). J. Am. Chem. Soc.

[5] Eaton PE, Lee CH, Xiong Y (1989). J. Am. Chem. Soc.

[6a] Krasovskiy A, Knochel P (2004). Angew. Chem.

[b01] (2004). Angew. Chem. Int. Ed.

[6b] Wunderlich SH, Rohbogner CJ, Unsinn A, Knochel P (2010). Org. Process Res. Dev.

[7a] Mulvey RE, Mongin F, Uchiyama M, Kondo Y (2007). Angew. Chem.

[b02] (2007). Angew. Chem. Int. Ed.

[7b] Mulvey RE (2009). Acc. Chem. Res.

[8] Lappert M, Protchenko A, Power P, Seeber A (2008). Metal Amide Chemistry.

[9a] Clegg W, Mulvey RE, Snaith R, Toogood GE, Wade K (1986). J. Chem. Soc. Chem. Commun.

[9b] Barr D, Clegg W, Mulvey RE, Snaith R (1989). J. Chem. Soc. Chem. Commun.

[9c] Barnett NDR, Mulvey RE, Clegg W, O’Neil PA (1992). Polyhedron.

[10a] Henderson KW, Williard PG, Bernstein PR (1995). Angew. Chem.

[b03] (1995). Angew. Chem. Int. Ed. Engl.

[10b] Clegg W, Liddle ST, Drummond AM, Mulvey RE, Robertson A (1999). Chem. Commun.

[10c] Armstrong DR, Clegg W, Drummond AM, Liddle ST, Mulvey RE (2000). J. Am. Chem. Soc.

[10d] Wei X, Dong Q, Tong H, Chao J, Liu D, Lappert MF (2008). Angew. Chem.

[b04] (2008). Angew. Chem. Int. Ed.

[11a] Clegg W, Henderson KW, Horsburgh L, McKenzie FM, Mulvey RE (1998). Chem. Eur. J.

[11b] Veith M, Wieczorek S, Fries K, Huch V (2000). Z. Anorg. Allg. Chem.

[12a] MacKenzie FM, Mulvey RE, Clegg W, Horsburgh L (1996). J. Am. Chem. Soc.

[12b] Holland R, Jeffery JC, Russell CA (1999). J. Chem. Soc. Dalton Trans.

[12c] Kennedy AR, MacLellan JG, Mulvey RE (2001). Angew. Chem.

[b05] (2001). Angew. Chem. Int. Ed.

[13] Baker DR, Mulvey RE, Clegg W, O’Neil PA (1993). J. Am. Chem. Soc.

[14] Tricotet T, Fleming P, Cotter J, Hogan A-ML, Strohmann C, Gessner VH, O’Shea DF (2009). J. Am. Chem. Soc.

[15] Fleming P, O’Shea DF (2011). J. Am. Chem. Soc.

[16a] Renaud P, Fox MA (1988). J. Am. Chem. Soc.

[16b] Romesberg FE, Harrison AT, Fuller DJ, Collum DB (1991). J. Am. Chem. Soc.

[16c] Romesberg FE, Collum DB (1992). J. Am. Chem. Soc.

[16d] Lucht BL, Collum DB (1994). J. Am. Chem. Soc.

[16e] Remenar JF, Lucht BL, Kruglyak D, Romesberg FE, Gilchrist JH, Collum DB (1997). J. Org. Chem.

[16f] Armstrong DR, García-Álvarez P, Kennedy AR, Mulvey RE, Robertson SD (2011). Chem. Eur. J.

[17a] Mulvey RE (2006). Organometallics.

[17b] Clegg W, Dale SH, Hevia E, Hogg LM, Honeyman GW, Mulvey RE, O’Hara CT, Russo L (2008). Angew. Chem.

[b06] (2008). Angew. Chem. Int. Ed.

[17c] Kennedy AR, Klett J, Mulvey RE, Wright DS (2009). Science.

[17d] Mulvey RE, Blair VL, Clegg W, Kennedy AR, Klett J, Russo L (2010). Nat. Chem.

[17e] Armstrong DR, Graham DV, Kennedy AR, Mulvey RE, O’Hara CT (2008). Chem. Eur. J.

[18] Williard PG, Nichols MA (1991). J. Am. Chem. Soc.

[19] Kottke T, Stalke D (1993). Angew. Chem.

[b001] (1993). Angew. Chem. Int. Ed. Engl.

[b002] Weiss E, Lambertsen T, Schubert B, Cockcroft JK, Weidemann A (1990). Chem. Ber.

[b003] Walfort B, Lameyer L, Weiss W, Herbst-Irmer R, Bertermann R, Rocha J, Stalke D (2001). Chem. Eur. J.

[20] Andrews PC, Barnett NDR, Mulvey RE, Clegg W, O’Neil PA, Barr D, Cowton L, Dawson AJ, Wakefield BJ (1996). J. Organomet. Chem.

[21] Gehrhus B, Hitchcock PH, Kennedy AR, Lappert MF, Mulvey RE, Rodger PJA (1999). J. Organomet. Chem.

[22] Lappert MF, Slade MJ, Singh A, Atwood JL, Rogers RD, Shakir R (1983). J. Am. Chem. Soc.

[23a] Barnett NDR, Mulvey RE, Clegg W, O’Neil PA (1991). J. Am. Chem. Soc.

[23b] Morris JJ, Noll BC, Henderson KW (2007). Acta Crystallogr. Sect. E.

[24] Allen FH (2002). Acta Crystallogr. Sect. B.

[25] Frisch MJ, Trucks GW, Schlegel HB, Scuseria GE, Robb MA, Cheeseman JR, Montgomery JA, Vreven T, Kudin KN, Burant JC, Millam JM, Iyengar SS, Tomasi J, Barone V, Mennucci B, Cossi M, Scalmani G, Rega N, Petersson GA, Nakatsuji H, Hada M, Ehara M, Toyota K, Fukuda R, Hasegawa J, Ishida M, Nakajima T, Honda Y, Kitao O, Nakai H, Klene M, Li X, Knox JE, Hratchian HP, Cross JB, Bakken V, Adamo C, Jaramillo J, Gomperts R, Stratmann RE, Yazyev O, Austin AJ, Cammi R, Pomelli C, Ochterski JW, Ayala PY, Morokuma K, Voth GA, Salvador P, Dannenberg JJ, Zakrzewski VG, Dapprich S, Daniels AD, Strain MC, Farkas O, Malick DK, Rabuck AD, Raghavachari K, Foresman JB, Ortiz JV, Cui Q, Baboul AG, Clifford S, Cioslowski J, Stefanov BB, Liu G, Liashenko A, Piskorz P, Komaromi I, Martin RL, Fox DJ, Keith T, Al-Laham MA, Peng CY, Nanayakkara A, Challacombe PMW, Gill B, Johnson W, Chen MW, Wong C, Gonzalez C, Pople JA

[26a] Hehre WJ, Ditchfield R, Pople JA (1972). J. Chem. Phys.

[26b] Hariharan PC, Pople JA (1973). Theor. Chim. Acta.

[27a] Kohn W, Becke AD, Parr RG (1996). J. Phys. Chem.

[27b] Becke AD (1988). Phys. Rev. A.

[27c] Lee CT, Yang W, Parr RG (1988). Phys. Rev. B.

[28a] McLean AD, Chandler GS (1980). J. Chem. Phys.

[28b] Krishnan R, Binkley JS, Seeger R, Pople JA (1980). J. Chem. Phys.

