# Supplementary material for: Developing a Hetero-Alkali-Metal Chemistry of 2,2,6,6-Tetramethyl-piperidide (TMP): Stoichiometric and Structural Diversity within a Series of Lithium/Sodium, Lithium/Potassium and Sodium/Potassium TMP Compounds
Source: Chemistry. 2011 Jul 15;17(32):8820–31. doi: 10.1002/chem.201101167 (PMC3761191; doi:10.1002/chem.201101167)
Supplement: Supplementary file 1 [file chem0017-8820-SD1.pdf]

CHEMISTRY
A EUROPEAN JOURNAL

# Supporting Information

© Copyright Wiley-VCH Verlag GmbH & Co. KGaA, 69451 Weinheim, 2011

## Developing a Hetero-Alkali-Metal Chemistry of 2,2,6,6-Tetramethylpiperidide (TMP): Stoichiometric and Structural Diversity within a Series of Lithium/Sodium, Lithium/Potassium and Sodium/Potassium TMP Compounds

**David R. Armstrong, Alan R. Kennedy, Robert E. Mulvey,* and Stuart D. Robertson*[a]**

chem_201101167_sm_miscellaneous_information.pdf

## Experimental

### General Experimental

All reactions and manipulations were carried out under a protective argon atmosphere using either standard Schlenk techniques or a glove box. All solvents were dried over Na/benzophenone and freshly distilled prior to use. $^{n}$BuLi (1.6 M in hexanes) was purchased from Aldrich and used as received. TMP(H) was purchased from Merck and stored over 4Å molecular sieves. TMEDA and PMDETA were distilled over $CaH_2$ and stored over 4Å molecular sieves prior to use. $^{n}$BuNa [1] and $KCH_2SiMe_3$ [2] were prepared by literature methods.

NMR spectra were recorded on a Bruker AV400 MHz spectrometer operating at 400.13 MHz for $^{1}$H, 155.47 MHz for $^{7}$Li and 100.62 MHz for $^{13}$C. All $^{13}$C NMR spectra were proton decoupled. Ion chromatography was carried out on a Dionex DX-100 Ion Chromatograph fitted with an IonPac CS12 column, an IonPac CG12A guard column and a CSRS – ULTRA 4mm suppressor. Flame Atomic Absorption Spectroscopy was carried out on a Perkin Elmer A-Analyst 200 Atomic Absorption Spectrometer. Elemental Analyses were performed by Denise Gilmour, University of Strathclyde Elemental Analysis Service.

Synthesis of $LiNa(TMP)_2$·TMEDA **2**

$^{n}$BuNa (0.126 g, 1.6 mmol) was suspended in hexane (5 mL) and sonicated. $^{n}$BuLi (1 mL, 1.6 M in hexanes, 1.6 mmol) was added and this was stirred for 30 mins. TMPH (0.54 mL, 3.2 mmol) was added followed by TMEDA (0.24 mL, 1.6 mmol) to give a clear yellow solution. This was immediately cooled to -35$^{o}$C overnight to yield a crop of pale yellow crystals (0.556 g, 82 %).

El. Analysis calc. for $C_{24}H_{52}LiN_4Na$ ($M_r$ = 426.63) C, 67.56; H, 12.29; N, 13.13; found: C, 66.44; H, 12.74; N, 12.70.

Synthesis of $[LiNa(TMP)_2]_8$ **3**

This was prepared in the same manner as **2** (vide supra) but rather than immediately cooling upon addition of TMEDA, the solution was stirred at room temperature until **3** crashed out as an insoluble pale powder. Crystals could be obtained by ceasing stirring before precipitation (approx 2 minutes) and leaving the solution to stand at room temperature for 24 - 48 hours.

El. Analysis calc. for $C_{18}H_{36}LiN_2Na$ ($M_r$ = 310.42) C, 69.64; H, 11.69; N, 9.02; found: C, 68.10; H, 11.82; N, 8.93.

Synthesis of $Li_2K(TMP)_3$·TMEDA **4**

$KCH_2SiMe_3$ (0.202 g, 1.6 mmol) was suspended in hexane and sonicated. $^nBuLi$ (2 mL, 1.6 mL in hexanes, 3.2 mmol) was added and this was stirred for 30 mins. TMPH (0.81 mL, 4.8 mmol) was added followed by TMEDA (0.24 mL, 1.6 mmol) to give a clear yellow solution. This was cooled to -35°C overnight to yield a crop of pale yellow crystals (0.609 g, 65 %).

$^1H$ NMR ($D_{12}$-cy): 2.31 (s, 4 H, TMEDA $CH_2$), 2.21 (s, 12 H, TMEDA $CH_3$), 1.68 (m, 6 H, TMP γ), 1.21 (t, $^2J_{H, H}$ = 6 Hz, TMP β), 1.18 (s, TMP $CH_3$), 1.10 (s, TMP $CH_3$). The final three resonances were broad and overlapped slightly, their combined integral was 44 H, 48 H expected for β + $CH_3$ of TMP.

$^{13}C$ NMR ($D_{12}$-cy): 58.5 (TMEDA $CH_2$), 52.9 (TMP α), 45.5 (TMEDA $CH_3$), 43.7 (TMP β), 37.7 (TMP $CH_3$), 20.8 (TMP γ).

$^7Li$ NMR ($D_{12}$-cy): 2.86 (broad singlet), 2.48 (small shoulder).

El. Analysis calc. for $C_{33}H_{70}Li_2KN_5$ ($M_r$ = 589.92) C, 67.19; H, 11.96; N, 11.87; found: C, 65.93; H, 12.60; N, 11.63.

Synthesis of $LiNa(TMP)_2$·PMDETA **5**

$^nBuNa$ (0.126 g, 1.6 mmol) was suspended in hexane (5 mL) and sonicated. $^nBuLi$ (1 mL, 1.6 M in hexanes, 1.6 mmol) was added and this was stirred for 30 mins. TMPH (0.54 mL, 3.2 mmol) was added followed by PMDETA (0.34 mL, 1.6 mmol) to give a clear yellow solution. This was cooled to -35$^o$C overnight to yield a crop of pale yellow crystals (0.731 g, 94 %).

$^1H$ NMR ($D_{12}$-cy): 2.42 (t, 4 H, $^2J_{H, H}$ = 6 Hz, $CH_2$ PMDETA), 2.35 (t, 4 H, $^2J_{H, H}$ = 6 Hz, $CH_2$ PMDETA), 2.27 (s, 3 H, $CH_3$ PMDETA), 2.24 (s, 12 H, $CH_3$ PMDETA), 1.71 (m, 4 H, $^2J_{H, H}$ = 6 Hz, $\gamma$ $CH_2$ TMP), 1.25 (br, 8 H, $\beta$ $CH_2$ TMP), 1.13 (br s, 24 H, $CH_3$ TMP).

$^{13}C$ NMR ($D_{12}$-cy): 58.7 (PMDETA $CH_2$), 56.4 (PMDETA $CH_2$), 52.7 (TMP $\alpha$), 46.6 (PMDETA $CH_3$ x 4), 44.5 (PMDETA $CH_3$), 42.8 (TMP $\beta$), 37.7 (TMP $CH_3$), 21.1 (TMP $\gamma$).

$^7Li$ NMR ($D_{12}$-cy): 2.81 (broad singlet).

El. Analysis calc. for $C_{27}H_{59}LiN_5Na$ ($M_r$ = 483.72) C, 67.04; H, 12.29; N, 14.48; found: C, 65.70; H, 12.28; N, 14.13.

Synthesis of $Li_2K(TMP)_3$·PMDETA **6**

$KCH_2SiMe_3$ (0.202 g, 1.6 mmol) was suspended in hexane and sonicated. $^nBuLi$ (2 mL, 1.6 mL in hexanes, 3.2 mmol) was added and this was stirred for 30 mins. TMPH (0.81 mL, 4.8 mmol) was added followed by PMDETA (0.34 mL, 1.6 mmol) to give a clear yellow solution. This was immediately cooled to -35$^o$C overnight where a

yellow oil precipitated from the colourless solvent. Warming to room temperature slowly crystallized a crop of pale yellow crystals (0.270 g, 26 %).

$^{1}$H NMR ($D_{12}$-cy): 2.41 (t, 4 H, $^{2}J_{H, H}$ = 6 Hz, $CH_2$ PMDETA), 2.36 (t, 4 H, $^{2}J_{H, H}$ = 6 Hz, $CH_2$ PMDETA), 2.26 (s, 3 H, $CH_3$ PMDETA), 2.24 (s, 12 H, $CH_3$ PMDETA), 1.70 (m, 6 H, γ $CH_2$ TMP), 1.23 (t, 12 H, $^{2}J_{H, H}$ = 6 Hz, β $CH_2$ TMP), 1.15 (s, 36 H, $CH_3$ TMP).

$^{13}$C NMR ($D_{12}$-cy): 58.1 (PMDETA $CH_2$), 56.3 (PMDETA $CH_2$), 52.8 (TMP α), 46.1 (PMDETA $CH_3$ x 4), 43.3 (PMDETA $CH_3$), 42.7 (TMP β), 37.6 (TMP $CH_3$), 21.2 (TMP γ).

$^{7}$Li NMR ($D_{12}$-cy): 2.85 (broad singlet), 2.48 (small shoulder).

El. Analysis calc. for $C_{36}H_{77}Li_2KN_6$ ($M_r$ = 647.02) C, 66.83; H, 12.00; N, 12.99; found: C, 66.49; H, 12.73; N, 12.88.

Synthesis of LiK(TMP)$_2$·PMDETA **7**

$^{n}$BuNa (0.126 g, 1.6 mmol) and $KCH_2SiMe_3$ (0.202 g, 1.6 mmol) were sonicated in hexane (10 mL). $^{n}$BuLi, (1 mL, 1.6 M in hexanes, 1.6 mmol) was added via syringe, followed by TMP(H) (0.81 mL, 4.8 mmol) to give a yellow suspension. PMDETA was added until a homogeneous solution resulted (0.66 mL, 3.2 mmol required). This was cooled to -35$^{o}$C overnight to yield a crop of colourless crystals (0.576 g, 72%).

$^{1}$H NMR ($D_{12}$-cy): 2.39 (t, 4 H, $^{2}J_{H, H}$ = 6 Hz, $CH_2$ PMDETA), 2.33 (t, 4 H, $^{2}J_{H, H}$ = 6 Hz, $CH_2$ PMDETA), 2.24 (s, 3 H, $CH_3$ PMDETA), 2.21 (s, 12 H, $CH_3$ PMDETA), 1.68 (m, 4 H, γ $CH_2$ TMP), 1.22 (t, 8 H, $^{2}J_{H, H}$ = 6 Hz, β $CH_2$ TMP), 1.09 (s, 24 H, $CH_3$ TMP).

$^{13}C$ NMR ($D_{12}$-cy): 58.3 (PMDETA $CH_2$), 56.5 (PMDETA $CH_2$), 52.8 (TMP α), 46.2 (PMDETA $CH_3$ x 4), 43.3 (PMDETA $CH_3$), 42.8 (TMP β), 37.8 (TMP $CH_3$), 21.3 (TMP γ).

$^7Li$ NMR ($D_{12}$-cy): 2.89 (broad singlet).

Satisfactory elemental analysis of **7** could not be obtained as the crystals were contaminated with a small amount of complex **8** as shown by X-ray crystallography.

Synthesis of NaK(TMP)$_2$·PMDETA **8**

$^n$BuNa (0.126 g, 1.6 mmol) and $KCH_2SiMe_3$ (0.202 g, 1.6 mmol) were sonicated in hexane (10 mL). TMP(H) (0.54 mL, 3.2 mmol) was added via syringe to give a yellow suspension. PMDETA was added until a homogeneous yellow solution resulted (0.66 mL, 3.2 mmol required). This was cooled to -35$^o$C overnight to yield a crop of pale yellow crystals (0.241 g, 29 %).

$^1H$ NMR ($D_{12}$-cy): 2.38 (t, 4 H, $^2J_{H, H}$ = 6 Hz, $CH_2$ PMDETA), 2.33 (t, 4 H, $^2J_{H, H}$ = 6 Hz, $CH_2$ PMDETA), 2.26 (s, 3 H, $CH_3$ PMDETA), 2.21 (s, 12 H, $CH_3$ PMDETA), 1.67 (m, 4 H, γ $CH_2$ TMP), 1.22 (t, 8 H, $^2J_{H, H}$ = 6 Hz, β $CH_2$ TMP), 1.05 (s, 24 H, $CH_3$ TMP).

$^{13}C$ NMR ($D_{12}$-cy): 58.4 (PMDETA $CH_2$), 56.8 (PMDETA $CH_2$), 53.2 (TMP α), 46.1 (PMDETA $CH_3$ x 4), 43.1 (PMDETA $CH_3$), 43.0 (TMP β), 38.4 (TMP $CH_3$), 21.6 (TMP γ).

El. Analysis calc. for $C_{27}H_{59}LiN_5Na$ ($M_r$ = 515.88) C, 62.86; H, 11.53; N, 13.58; found: C, 62.55; H, 11.77; N, 13.94.

**Crystallographic data** was collected at 123(2)K on Oxford Diffraction instruments using either $Cu_{K\alpha}$ ($l$ = 1.54180Å, **2**) or $Mo_{K\alpha}$ ($l$ = 0.71073Å, **3-8**) radiation. Structures were solved using *SHELXS-97* and refined to convergence against $F^2$

against all independent reflections by the full-matrix least-squares method using the *SHELXL-97* program.[3] Refining a model for **3** was complicated by Li and Na atoms sharing sites (mutual substitution disorder). For each crystallographically independent metal site, the Li and Na sofs were refined with the total occupancy of Li + Na set to 1 and the Li/Na pair constrained to have equal atomic coordinates and anisotropic displacement parameters (EXYZ & EADP). This gave an overall Li:Na ratio of 57:43 – in line with the results of other characterisation. Due to the size difference between Li and Na, the ligand positions were also disordered. Due to lack of observed data, this was only modelled where a significant change in ligand position was observed.

| | **2** | **3** | **4** | **5** | **6** | **7** | **8** |
|---|---|---|---|---|---|---|---|
| empirical formula | $C_{24}H_{52}LiN_4Na$ | $C_{36}H_{72}Li_{2.28}N_4Na_{1.72}$ | $C_{33}H_{70}KLi_2N_5$ | $C_{27}H_{59}LiN_5Na$ | $C_{36}H_{77}KLi_2N_6$ | $C_{27}H_{59}KLi_{0.90}N_5Na_{0.10}$ | $C_{27}H_{59}KN_5Na$ |
| $M_r$ | 426.63 | 616.34 | 589.92 | 483.72 | 647.02 | 501.49 | 515.88 |
| crystal system | Orthorhombic | Monoclinic | Monoclinic | Triclinic | Monoclinic | Monoclinic | Monoclinic |
| space group | Fdd2 | P 21/c | C 2/c | P-1 | P 21/n | P 21/m | P 21/m |
| *a* [Å] | 29.1028(19) | 15.3672(10) | 12.4647(3) | 10.4133(4) | 11.3844(2) | 10.1233(6) | 9.9788(5) |
| *b* [Å] | 17.4203(17) | 11.4925(12) | 20.2542(4) | 11.3512(5) | 19.4595(4) | 15.2831(9) | 15.2035(6) |
| *c* [Å] | 10.7761(9) | 22.2734(17) | 15.9718(5) | 14.9896(7) | 18.9186(5) | 10.7052(6) | 10.8898(5) |
| *a* [$^o$] | 90 | 90 | 90 | 73.989(4) | 90 | 90 | 90 |
| *b* [$^o$] | 90 | 95.176(6) | 109.549(3) | 71.957(4) | 96.739(2) | 108.727(6) | 108.100(5) |
| *g* [$^o$] | 90 | 90 | 90 | 71.699(4) | 90 | 90 | 90 |
| *V* [Å$^3$] | 5463.3(8) | 3917.6(6) | 3799.84(18) | 1567.88(12) | 4162.17(16) | 1568.58(16) | 1570.37(12) |
| *Z* | 8 | 4 | 4 | 2 | 4 | 2 | 2 |
| $r_{calcd}$ [g cm$^{-3}$] | 1.037 | 1.045 | 1.031 | 1.025 | 1.033 | 1.062 | 1.091 |
| reflns measured | 10248 | 24958 | 18146 | 17365 | 19116 | 6849 | 7177 |
| unique reflns | 2548 | 6894 | 5034 | 8063 | 9299 | 3797 | 3808 |
| R int | 0.1763 | 0.0831 | 0.0349 | 0.0524 | 0.0281 | 0.0254 | 0.0362 |

| | | | | | | | |
|---|---|---|---|---|---|---|---|
| GooF | 0.919 | 0.897 | 0.960 | 0.950 | 1.025 | 1.051 | 1.033 |
| *R* [on *F* , obs rflns only] | 0.0679 | 0.0854 | 0.0407 | 0.0568 | 0.0538 | 0.0510 | 0.0563 |
| *wR* [on $F^2$,all data] | 0.1796 | 0.2422 | 0.0982 | 0.1228 | 0.1253 | 0.1082 | 0.1251 |
| Largest diff. peak/hole [eÅ$^{-3}$] | 0.359/-0.322 | 0.546/-0.461 | 0.257/-0.210 | 0.266/-0.226 | 0.242/-0.218 | 0.272/-0.294 | 0.302/-0.454 |

**Table S1** Crystallographic data and refinement details for compounds **2-8**.

## DFT Calculations

### Energetics of Li/Na TMEDA system:

¼$(LiTMP)_4$ + ½$(TMEDA{\cdot}NaTMP)_2$ = TMEDA·Na(μ-TMP)$_2$Li

? E = -1.63 kcal mol$^{-1}$

½$(LiTMP)_4$ + ½$(TMEDA{\cdot}NaTMP)_2$ = TMEDA·Na(μ-TMP)$_3$Li$_2$

? E = -0.10 kcal mol$^{-1}$

### Energetics of Li/K TMEDA system:

¼$(LiTMP)_4$ + ½$(TMEDA{\cdot}KTMP)_2$ = TMEDA·K(μ-TMP)$_2$Li

? E = -0.40 kcal mol$^{-1}$

½$(LiTMP)_4$ + ½$(TMEDA{\cdot}KTMP)_2$ = TMEDA·K(μ-TMP)$_3$Li$_2$

? E = -3.25 kcal mol$^{-1}$

### Energetics of Na/K TMEDA system:

½$(TMEDA{\cdot}NaTMP)_2$ + ½$(TMEDA{\cdot}KTMP)_2$ = TMEDA·K(μ-TMP)$_2$Na·TMEDA

? E = +0.42 kcal mol$^{-1}$

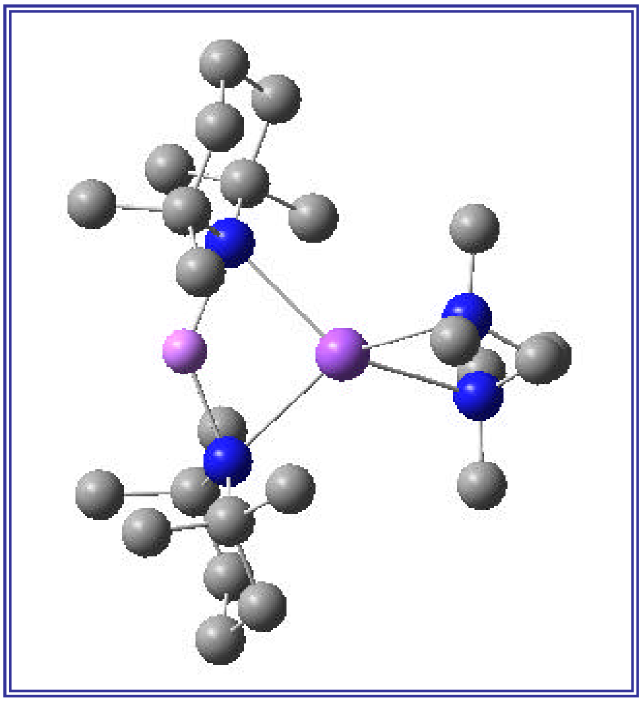

**Figure S1** Calculated structure of complex **2**

| | |
|---|---|
| Li-$N_{TMP}$ | 1.941 |
| Na-$N_{TMEDA}$ | 2.630 |
| Na-$N_{TMP}$ | 2.592 |
| $N_{TMP}$-Li-$N_{TMP}$ | 136.4 |
| $N_{TMP}$-Na-$N_{TMP}$ | 88.1 |
| Li-$N_{TMP}$-Na | 67.8 |

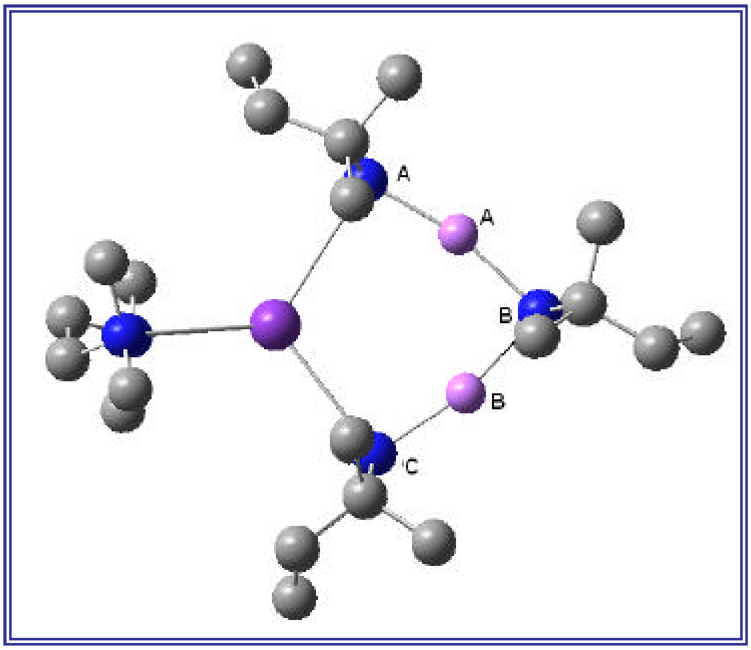


**Figure S2** Calculated structure of complex **4**

| | |
|---|---|
| $Li_A$-$N_A$ | 1.941 |
| $Li_A$-$N_B$ | 2.029 |
| $Li_B$-$N_B$ | 1.999 |
| $Li_B$-$N_C$ | 1.988 |
| K-$N_A$ | 3.087 |
| K-$N_C$ | 2.951 |
| K-$N_{TMEDA}$ | 3.119, 3.121 |
| $N_A$-$Li_A$-$N_B$ | 166.5 |
| $N_B$-$Li_B$-$N_C$ | 165.7 |
| K-$N_A$-$Li_A$ | 92.2 |
| $Li_A$-$N_B$-$Li_B$ | 91.4 |
| $Li_B$-$N_C$-K | 94.0 |
| $N_A$-K-$N_C$ | 110.2 |

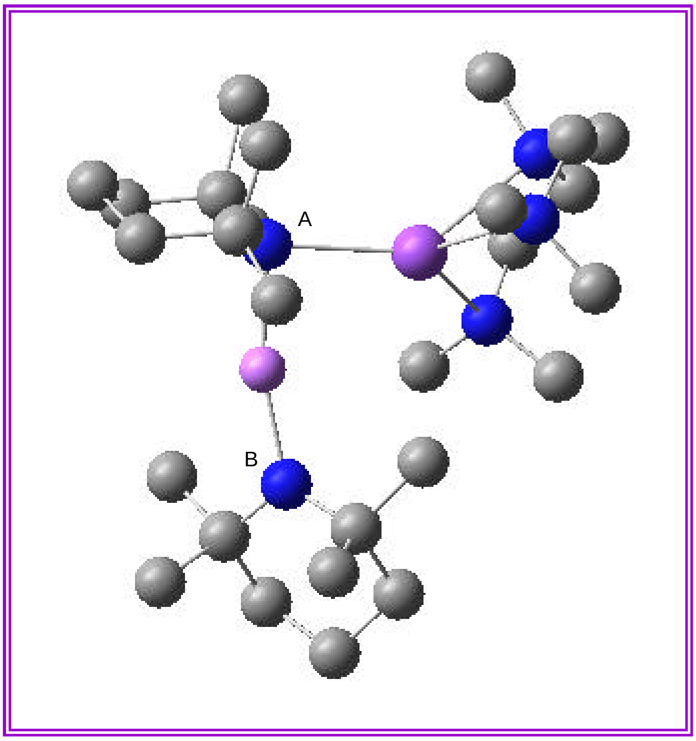


**Figure S3** Calculated structure of complex **5**

| | |
|---|---|
| Li-$N_A$ | 2.008 |
| Li-$N_B$ | 1.877 |
| Na-$N_A$ | 2.465 |
| Na-$N_{PMDETA}$ | 2.651, 2.694, 2.756 |
| $N_A$-Li-$N_B$ | 166.2 |
| Na-$N_A$-Li | 87.5 |

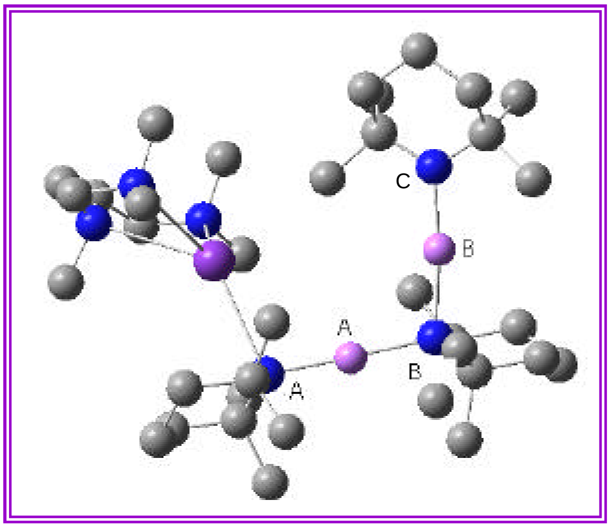


**Figure S4** Calculated structure of complex **6**

| | |
|---|---|
| K-$N_A$ | 2.919 |
| $Li_A$-$N_A$ | 1.964 |
| $Li_A$-$N_B$ | 1.983 |
| $Li_B$-$N_B$ | 2.033 |
| $Li_B$-$N_C$ | 1.881 |
| K-$N_{PMDETA}$ | 3.059, 2.997, 3.027 |
| K-$N_A$-$Li_A$ | 71.8 |
| $N_A$-$Li_A$-$N_B$ | 102.9 |
| $Li_A$-$N_B$-$Li_B$ | 99.6 |
| $N_B$-$Li_B$-$N_C$ | 171.0 |

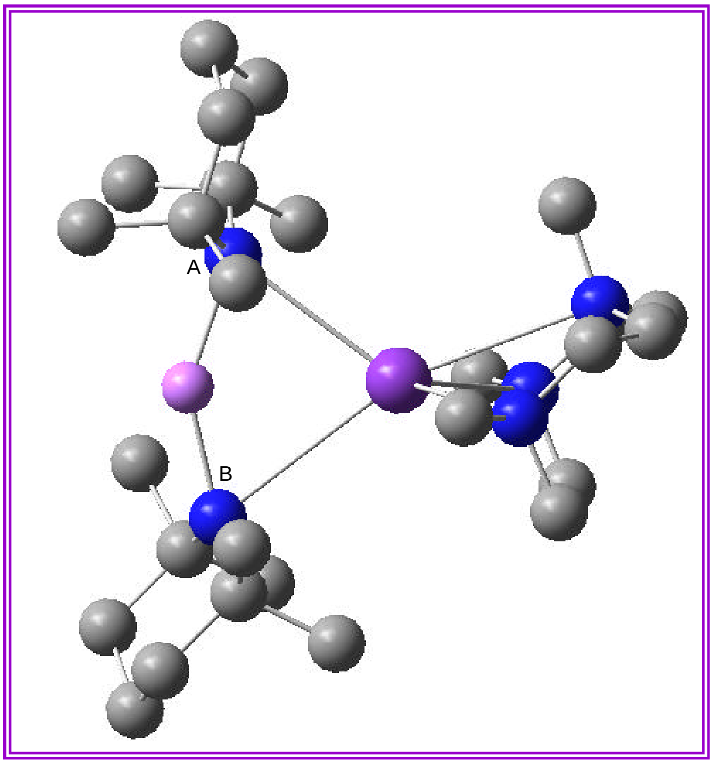


**Figure S5** Calculated structure of complex **7**

| | |
|---|---|
| Li-$N_A$ | 1.991 |
| Li-$N_B$ | 1.919 |
| K-$N_A$ | 2.924 |
| K-$N_B$ | 3.237 |
| K-$N_{PMDETA}$ | 3.142, 3.049, 3.095 |
| $N_A$-Li-$N_B$ | 148.3 |
| K-$N_A$-Li | 71.8 |
| K-$N_B$-Li | 64.9 |

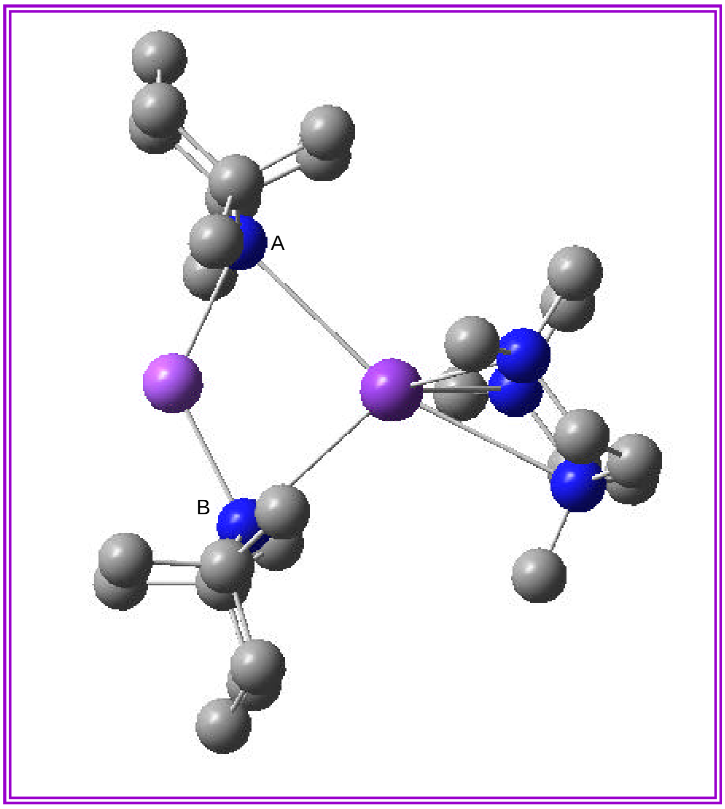


**Figure S5** Calculated structure of complex **8**

| | |
|---|---|
| Na-$N_A$ | 2.300 |
| Na-$N_B$ | 2.355 |
| K-$N_A$ | 3.129 |
| K-$N_B$ | 2.957 |
| K-$N_{PMDETA}$ | 3.123 3.115 3.074 |
| $N_A$-Na-$N_B$ | 128.2 |
| $N_A$-K-$N_B$ | 86.9 |
| K-$N_A$-Na | 71.0 |
| K-$N_B$-Na | 73.8 |

## References


[1] C. Schade, W. Bauer, P.v.R. Schleyer, *J. Organomet. Chem.* **1985**, *295*, C25.

[2] B. Conway, D.V. Graham, E. Hevia, A.R. Kennedy, J. Klett, R.E. Mulvey, *Chem. Commun.* **2008**, 2638.

[3] G.M. Sheldrick, *Acta Cryst.* **2008**, *A64*, 112.
